# Physicians’ experiences, attitudes, and beliefs towards medical cannabis: a systematic literature review

**DOI:** 10.1186/s12875-021-01559-w

**Published:** 2021-10-21

**Authors:** Sabrina Trappaud Rønne, Frederik Rosenbæk, Line Bjørnskov Pedersen, Frans Boch Waldorff, Jesper Bo Nielsen, Helle Riisgaard, Jens Søndergaard

**Affiliations:** 1 Research Unit of General Practice, Department of Public Health, University of Southern Denmark, J.B. Winsløws Vej 9A, 5000 Odense C, Denmark; 2 DaCHE – Danish Centre for Health Economics, Department of Public Health, University of Southern Denmark, Winsløws Vej 9B, 5000 Odense C, Denmark; 3 The Research Unit for General Practice and Section of General Practice, Department of Public Health, University of Copenhagen, Copenhagen, Denmark

**Keywords:** Medical cannabis, Physicians, Attitudes, Experiences, Barriers, Facilitators

## Abstract

**Background:**

An increasing number of countries legalise the use of medical cannabis or allow it for a narrow range of medical conditions. Physicians, and often the patients’ general practitioner, play a major role in implementing this policy. Many of them, however, perceive a lack of evidence-based knowledge and are not confident with providing patients with medical cannabis. The objectives of this review are to synthesise findings about hospital physicians’ and GPs’ experiences, attitudes, and beliefs towards the use of medical cannabis with the purpose of identifying barriers and facilitators towards providing it to their patients.

**Methods:**

Peer-reviewed articles addressing hospital physicians’ and GPs’ experiences, attitudes, and beliefs towards the use of medical cannabis were searched systematically in PubMed, Scopus, EMBASE, and the Cochrane Library.

**Results:**

Twenty-one articles were included from five different countries in which the medical cannabis laws varied. The studied physicians experienced frequent inquiries about medical cannabis from their patients (49–95%), and between 10 and 95% of the physicians were willing to prescribe and/or provide it to the patients, depending on setting, specialty and experience among the physicians. This review found that physicians experienced in prescribing medical cannabis were more convinced of its benefits and less worried about adverse effects than non-experienced physicians. However, physicians specialized in addiction treatment and certain relevant indication areas seemed more sceptical compared to physicians in general. Nevertheless, physicians generally experienced a lack of knowledge of clinical effects including both beneficial and adverse effects.

**Conclusion:**

This review indicates that GPs and hospital physicians from various specialties frequently experience patient demands for medical cannabis and to some degree show openness to using it, although there was a wide gap between studies in terms of willingness to provide. Hospital physicians and GPs’ experienced in prescribing are more convinced of effects and less worried of adverse effects. However, most physicians experience a lack of knowledge of beneficial effects, adverse effects and of how to advise patients, which may comprise barriers towards prescribing. More research, including larger studies with cohort designs and qualitative studies, is needed to further examine facilitators and barriers to physicians’ prescribing practices.

## Background

In recent years, some countries, including the Netherlands, Italy, Canada, Israel, Australia, and a number of states in the US, have legalized medical use of cannabis when prescribed or provided by healthcare professionals. In most of these countries, the cannabis is dispensed through pharmacies [1]. Other countries, including Denmark, Norway, Sweden, Poland, and the United Kingdom allow treatment with medical cannabis for a narrow range of medical conditions in patients where all other options of conventional treatment have been tried without reaching treatment targets [1, 2]. Most commonly, a specialist with a specific license prescribes the cannabis products, and also pharmacies need a license to supply them [1].

Medical cannabis has been debated worldwide among physicians and decision-makers, and the use of it remains controversial [3]. Moreover, the quality of the evidence of potential benefit as well as adverse effects is low [4, 5]. Furthermore, cannabis contains tetrahydrocannabinol (THC) which is the euphoric component in cannabis for recreational use [6], and for this reason it gives rise to concerns about abuse and addiction [5, 7]. However, medical cannabis and cannabis for recreational use are different from each other, as medical cannabis is subject to stricter requirements than recreational cannabis in terms of therapeutic safety, cultivation and manufacturing [6]. Another main component used in some preparations of medical cannabis is cannabidiol (CBD) which is non-euphoric. Depending on the needs to address, THC and CBD is given to patients in controlled, carefully metered doses [8].

Generally, physicians play a major role in implementing regulatory policies on the use of medical cannabis, and specifically general practitioners (GPs) who are often the patients’ first contact in healthcare systems and an ongoing coordinator of their treatment [9]. A recent systematic review study reviewed the existing literature concerning all types of health care professionals’ personal beliefs, knowledge, and concerns regarding delivery and supply of medical cannabis to patients [10]. However, it is just as essential to focus on physicians’ experiences with patients’ demand for medical cannabis and whether they decide to provide it to them [11].

Hence, in order to fill this knowledge gap, the objectives of this review were to investigate hospital physicians’ and GPs’ experiences with patients’ demand for medical cannabis and prescription practice, as well as their attitudes, and beliefs towards the use of medical cannabis with the purpose of identifying barriers and facilitators towards providing it to their patients.

## Methods

In this review we followed the guidelines given by the Preferred Reporting Items for Systematic Review and Meta-Analyses (PRISMA) Statement [12]. Peer-reviewed articles addressing hospital physicians’ and GPs’ experiences, attitudes, and beliefs towards medical cannabis were searched in the databases PubMed, Scopus, EMBASE, and the Cochrane Library. We searched databases through February 2019. The search strategy included search terms listed in Table 1.

**Table 1:** Search strategy (PubMed)

Variable	Search terms
Medical cannabis	*(“medical cannabis” OR “medical marihuana” OR “medical cannabidiol” OR “medical cannabinoids” OR “medicinal cannabis” OR “medicinal marihuana” OR “medicinal cannabidiol” OR “medicinal cannabinoids” OR “medical use of cannabis” OR “medical use of marijuana” OR “medical use of marihuana” OR “medical use of cannabidiol” OR “medical use of cannabinoids” OR “medicinal use of cannabis” OR “medicinal use of marijuana” OR “medicinal use of marihuana” OR “medicinal use of cannabidiol” OR “medicinal use of cannabinoids” OR “clinical use of cannabis” OR “clinical use of marijuana” OR “clinical use of marihuana” OR “clinical use of cannabidiol” OR “clinical use of cannabinoids” OR “therapeutic use of cannabis” OR “therapeutic use of marijuana” OR “therapeutic use of marihuana” OR “therapeutic use of cannabidiol” OR “therapeutic use of cannabinoids” OR “Prescription of cannabis” OR “prescription of marijuana” OR “prescription of marihuana” OR “prescription of cannabidiol” OR “prescription of cannabinoids” OR “ordination of cannabis” OR “ordination of marijuana” OR “ordination of marihuana” OR “ordination of cannabidiol” OR “ordination of cannabinoids”) AND*
Experiences, attitudes, and beliefs	*(Attitude OR attitudes OR position OR positions OR stance OR stances OR perspective OR perspectives OR view OR views OR opinion OR opinions OR belief OR beliefs OR experience OR experiences OR perception OR perceptions OR practice OR practices OR confidence OR needs OR barriers OR comfort) AND*
Physicians	*(physician OR “family practice” OR “family practitioner” OR providers OR “medical practitioner” OR “medical practice” OR “general practitioner” OR “general practice” OR doctor)*

The search strategy was adjusted for each specific database and its search mechanisms. Criteria for inclusion and exclusion are shown in Table 2. Articles were included if they, addressed physicians’ experiences, attitudes, or beliefs towards use of medical cannabis, were written in English, Danish, Norwegian or Swedish and published after year 2000. Articles were excluded if they only examined the use of medical cannabis in children. Besides, no exclusion criteria on the type of medical cannabis products were applied, and therefore, this review defines medical cannabis as both magistral preparations and herbal products. Magistral preparations are medical drugs with active ingredients of the cannabis plant, compounded in pharmacies and prescribed by the doctor to specific patients who do not benefit sufficiently from authorized medicines [6].

**Table 2:** Inclusion criteria

**Articles were included in this review, if they;**	
Addressed physicians’ experiences, attitudes, or beliefs towards use of medical cannabis. - We included studies that addresses both physicians and other health care professionals and/or patients, if we could extract results for physicians or if 50% or more of the study population were physicians. - We included studies if they addressed medical cannabis by involvement of physicians in the identification of the perceived need and use of medical cannabis on the patients.	
Were written in English, Danish, Norwegian or Swedish - Because we were interested in international peer-reviewed scientific articles, we included articles in the Scandinavian languages as well, because all authors were able to understand it.	
Were published after year 2000 - The year was chosen as a pragmatic cut off, by which articles would be discarded, due to contextual differences in the use of medical cannabis among health professionals and in the public. Research activity within this topic is new and current.	
**Articles were excluded from this review, if they;**	
Only examined the use of medical cannabis in children (< 18 years) - Children are physiologically different from adults, and the use of medical cannabis in their treatment may have severe consequences [13]. Hence, the beliefs and attitudes of physicians, regarding use of medical cannabis, may differ depending on the age of the population under investigation.	

**Fig. 1:**
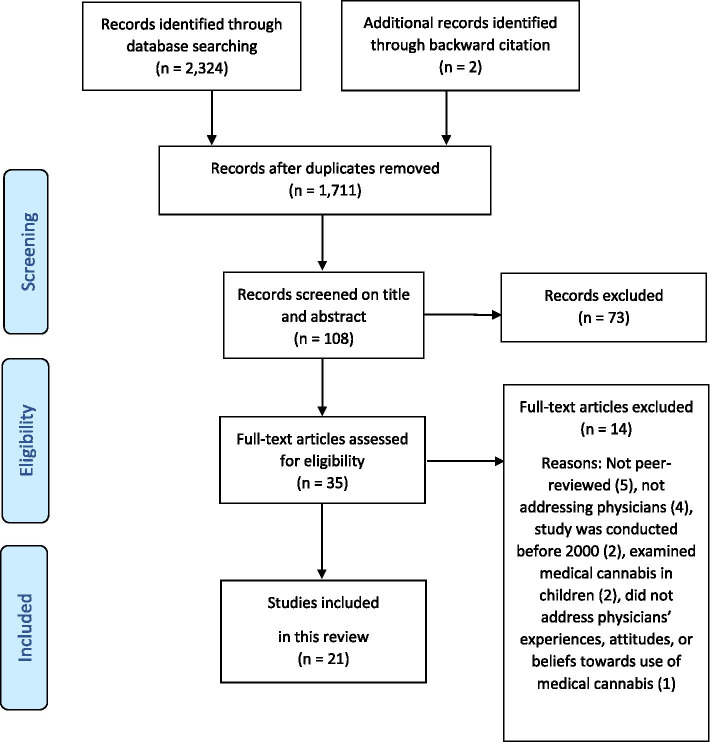
Flow chart illustrating selection of papers included in review [14]

Title and abstract were independently screened by one author, based on the stated exclusion criteria. The remaining articles were screened in full text by two authors. Disagreements were resolved by consensus or consulting with a senior researcher. Details of the literature search are shown in a flow diagram in Fig. 1.

## Results

### Characteristics of identified literature

This review includes 21 studies in total; 19 quantitative survey studies using questionnaires and two qualitative studies using open-ended questionnaires and interviews. All the identified studies aimed to explore experiences and/or attitudes among physicians towards the use of medical cannabis, and about half of the studies also investigated their perceived knowledge or educational needs regarding medical cannabis. Some studies included multiple types of physicians while other studies only included one or few specialties. The studies were conducted in different countries, however most studies were conducted in the United States (see Table 3).

**Table 3:** Study characteristics

	Number of studies
**Study methods**	
Qualitative studies using interviews and open-ended questions	2
Quantitative studies using questionnaires	19
**Country**	
United States	9
Canada	3
Israel	4
Ireland	2
Australia	3
**Study population**	
Physicians with various specialties	7
General practitioners	7
Rheumatologists	2
Neurologists	1
Oncologists	1
Psychiatrists	1
Pain specialists	1
Dermatologists	1
**Number of study subjects (physicians)**	
≤ 50	6
51–200	6
> 200	9

In the studies included, patient groups differed, and the laws on the use of medical cannabis varied. The literature details are shown in Table 4. Due to the heterogeneity in study populations as well as between patient groups and legislation, it was not possible to conduct a formal meta-analysis. Instead, we conducted a literature review, and the study results are presented as text.

**Table 4:** Literature details

Reference	Country	Objective	Study population and methods	Results/conclusion	Comments
Philpot 2019 [15]	USA (Minnesota)	To explore the attitudes, beliefs, and knowledge of healthcare providers in order to identify ongoing barriers, biases and knowledge gaps relating to medical cannabis.	62 providers answered a questionnaire (response rate: 31%). 76% of respondents were physicians.	Experiences: 27.4% was registered to certify patients for medical cannabis, but one half reported having patients who had been certified for medical cannabis. 10% of providers had been certifying patients, and the mean number of patients certified was 4. Attitudes and beliefs: 58.1% of the providers believed that medical cannabis was a legitimate medical therapy, and 38.7% believed that providers should be offering it to patients for managing medical conditions. 50% of providers were not ready to or did not want to answer patient questions about medical cannabis, and 77.4% of providers were interested in learning more about it. A majority (> 50%) of providers believed that medical cannabis was helpful for treating the qualifying medical conditions of cancer, terminal illness, and intractable pain. A majority of providers did not know if medical cannabis was effective for managing nearly one-half of the other state designated qualifying medical conditions. Few believed that medical cannabis improved quality of life domains. 38,7% of providers believed that medical cannabis interacted with medical therapies.	Minnesota has created a medical cannabis comprehensive program, and enrolled patients has access to extracted cannabis products in liquid or oil form.
Braun 2018 [16]	USA	To explore medical oncologists’ attitudes and knowledge in relation to prescription of medical cannabis and to explore its comparative effectiveness and risks.	237 medical oncologists answered a questionnaire (response rate: 63%). The study explored whether oncologists reported discussing medical marijuana with patients, recommended it clinically and felt sufficiently informed to make such recommendations. The study also explored medical marijuana’s comparative effectiveness for several conditions and its risks compared with prescription of opioids.	Experiences: 80% conducted discussions about MM with patients or their families, and 46% recommended MM clinically. Attitudes and beliefs: 30% of oncologists felt sufficiently informed to make recommendations regarding MM. More than half of oncologists who reported recommending MM did not consider themselves to have sufficient knowledge to make a recommendation. 67% viewed it as a helpful adjunct to standard pain management strategies, and 65% thought MM is equally or more effective than standard treatments for anorexia and cachexia.	It was legal for physicians to prescribe medical cannabis in 30 states and the District of Columbia.
Luba 2018 [17]	USA (46 states)	To examine palliative and hospice care providers’ attitudes and beliefs regarding the use of cannabis for terminally ill populations.	426 palliative and hospice care providers, (345 medical doctors, 58 nurses, and 23 others) answered a questionnaire (response rate was not reported).	Attitudes and beliefs: 70% of all participants reported that cannabis could aid in end-of-life care. When examining individual symptoms, almost 90% believed that cannabis could be helpful in treating nausea, appetite loss and pain, almost 70% believed it could be helpful in the treatment of sleep problems, almost 60% in the treatment of irritability and emotional suffering. More than half of the study participants believed that adverse effects of cannabis would be equally or less problematic than conventional treatments when considering pain, appetite loss, nausea, sleep, and end-of-life care. 61% of all participants would recommend cannabis to patients with a terminal illness. More than half would recommend it for the treatment of appetite loss, nausea and pain.	Respondents were from 46 different states. 45.3% of respondents were from states where cannabis was illegal, 36.2% were from states where cannabis was legal for medical use, and 22.1% were from states where cannabis was legal for recreational use. No further details on the prescription procedure or law on medical cannabis in each state were described.
Robinson 2018 [18]	USA	To explore the extent of dermatologists’ familiarity with and interest in cannabinoids as therapeutics.	531 dermatologists answered a questionnaire (response rate: 21.3%).	Experiences: 55% reported at least one patient-initiated discussion about cannabinoids in the last year. Attitudes and beliefs: 86% thought cannabinoids should be legal for medical treatment. Around 94% believed research into dermatologic uses of cannabinoids is worthwhile. 86% were willing to prescribe an approved cannabinoid as a topical treatment, and 71% were willing to prescribe an oral form. 64% did not know that cannabidiol is not psychoactive, and 29% did not know that tetrahydrocannabinol is psychoactive.	See the chapter “Procedures and definitions of medical cannabis prescriptions in included studies”.
Sideris 2018 [19]	USA (New York)	To probe physicians’ knowledge and perspectives of the New York State Medical Marijuana Programme (NY-MMP) and cannabinoids in patient care.	164 physicians with various specialties answered a questionnaire (response rate was not reported). 13% of the physicians were registered with the MMP, which authorized them to recommend medial cannabis.	Experiences: 95% of registered physicians and 49% of nonregistered physicians had patients who inquired about medical cannabis within the past year. More than 75% reported having patients who used cannabis for symptom control. 25% of all respondents had recommended synthetic cannabinoid drugs, which were approved by the Food and Drug Administration, for patients. Attitudes and beliefs: 71% believed that medical cannabis should be an option available to patients. 55% reported a willingness to discuss medical cannabis with patients if they felt their patients could benefit.	Medical cannabis was legal in the state of New York; Physicians can certify that patients have a qualifying condition and may recommend medical cannabis but cannot issue a prescription. To recommend medical cannabis, physicians must be registered with the NY-MMP, including a 4-h course which provides an overview of adverse effects, guidelines for dosing and administration etc.
Bega 2017 [20]	USA (14 states)	To gather data on cannabis-related prescribing practices and views regarding potential risks and benefits of medical cannabis among neurologists caring for patients with Parkinson’s disease.	56 neurologists answered a questionnaire (response rate: 63%).	Experiences: 80% of all respondents reported having patients using medical cannabis (not described if they used it legally or illegally), and 95% had been requested to prescribe it. 10% of all respondents had recommended the use of cannabis to their patients. 23% reported having formal education on medical cannabis (not defined). Attitudes and beliefs: 93% expected medical cannabis to be effective for appetite loss, 85% for pain, 80% for nausea, and 67% for anxiety. More than half expected that medical cannabis would worsen motivation, sleepiness, balance, forgetfulness, and hallucinations.	Medical cannabis was legal in eight of the surveyed states and illegal in six of the states. No further details of the law on medical cannabis were described.
Mathern 2015 [21]	USA (study population was primarily from North America and Europe)	To study opinions about the use of medical cannabis for people with epilepsy among physicians, other health professionals and patients/the public. (Only results from physicians are included in this review).	609 (140 epileptologists, 31 general neurologists, 9 general physicians, 36 basic researchers, 40 nurses and 353 patients/public) from different countries answered a questionnaire (response rate not reported as participants were not invited individually). Only results on health professionals are presented in this review. The study compared epileptologists/neurologists and other physicians.	Attitudes and beliefs: Fewer epileptologists/neurologists supported the use of medical cannabis in the treatment of people with epilepsy compared with general medical personal.	Law on medical cannabis was not described. See the chapter “Procedures and definitions of medical cannabis prescriptions in included studies”.
Kondrad 2013 [22]	USA (Colorado)	To study physicians’ attitudes towards medical cannabis.	520 general practitioners (GPs) answered a questionnaire (response rate of 30%). The study compared physicians with and without experiences of prescribing medical cannabis to patients.	Experiences: 31% had ever recommended medical cannabis, primary to treat pain, muscle spasms and nausea. GPs reported a need for continuing medical education about medical cannabis. Attitudes and beliefs: Those who had recommended medical cannabis were significantly more likely to be convinced of its benefits and less concerned about its risks than physicians who had never recommended it (72% vs. 26% respectively (*p* < 0.001)).	In Colorado, physicians could certify a patient registry card allowing patients to use cannabis for medical conditions.
Charuvastra 2005 [23]	USA	To investigate physicians’ practices and attitudes regarding the use of medical cannabis.	960 physicians (various specialities; addiction medicine/psychiatry, family practice, general psychiatry, internal medicine, obstetrics and gynaecology) answered a questionnaire (response rate: 66%). The study compared physicians specialized in addiction medicine with other specialties.	Attitudes and beliefs: 36% reported that physicians should be able to prescribe medical cannabis legally. Compared to physicians specialized in addiction medicine (referent), internists and obstetrician/gynaecologists were significantly more likely to support medical use of cannabis, OR: 1.62 (1.01–2.50) and OR: 2.21 (1.39–3.53 respectively).	Law on medical cannabis was not described. See the chapter “Procedures and definitions of medical cannabis prescriptions in included studies”.
Jacobs 2018	Australia	To assess Australian psychiatrists’ and psychiatry trainees’ knowledge about and attitudes towards medicinal cannabinoids, given the recent relaxation of cannabinoid-prescribing laws in Australia.	88 physicians responded to an online accessible survey, with 55 completing all items, 23 psychiatrists and 32 trainees (response rate: 1.1%).	Attitudes and beliefs: 54% of respondents would prescribe medical cannabinoids if it was legal for them to do so (trainees did not have the authority). Participants believed there was evidence for the use of cannabidiol and tetrahydrocannabinol in treating childhood epilepsy, chronic pain, and nausea and vomiting. Generally, they were poor at differentiating between indications for cannabidiol versus tetrahydrocannabinol. They were most concerned about medicinal cannabinoids leading to psychotic symptoms, addiction and dependence, apathy and recreational use.	In Australia there was legal access to medical cannabis for patients approved for it by a physician.
Karanges 2018 [24]	Australia	To examine the knowledge and attitudes of Australian GPs towards medical cannabis, including patient demands, GP perceptions of therapeutic effects and potential harms, perceived knowledge and willingness to prescribe.	640 GPs answered a questionnaire (response rate 37%). Distribution of printed questionnaires at one-day general practice educational seminars held in five major Australian Cities.	Experience: (61.5%) reported one or more patient enquiries about medical cannabis in the last 3 months. Attitudes and beliefs: Most felt that their own knowledge was inadequate and only 28.8% felt comfortable discussing medical cannabis with patients. Over half (56.5%) supported availability on prescription, with the preferred access model involving trained GPs prescribing independently of specialists. Support for use of medical cannabis was condition-specific, with strong support for use in cancer pain, palliative care and epilepsy, and much lower support for use in depression and anxiety.	In Australia there was legal access to medical cannabis for patients approved for it by a physician.
Irvine 2006 [25]	Australia	To examine GPs’ attitudes to and knowledge of medical cannabis.	32 GPs answered a questionnaire (response rate not reported).	Attitudes and beliefs: 75% of the sample would be prepared to prescribe medical cannabis if it was legal, supported by peers and based on good-quality clinical research evidence.	In Australia, medical cannabis was illegal at the time.
St-Amant 2015 [26]	Canada (Quebec)	To measure prevalence and identified determinants of cannabinoid prescriptions for the management of chronic noncancer pain among physicians.	166 physicians (family physicians/GPs and other specialties) answered a questionnaire (response rate of 52.2%).	Experiences: 27% had prescribed cannabinoids for all potential indications in the previous year. 23% had prescribed it for chronic noncancer pain, and of these 18% were medical cannabis. The majority had prescribed it to less than five patients. Physicians were not comfortable prescribing cannabinoids, with about 80% reporting a degree of comfort below 6 on a 1–10-point scale. Physicians reported medical education and having guidelines and more clinical data as factors which could increase their comfort level. Low level of comfort with prescribing medical cannabis for chronic noncancer pain was associated with low prevalence of prescription (OR: 1.25, CI: 1,01-1,55).	See the chapter “Procedures and definitions of medical cannabis prescriptions in included studies”.
Ziemianski 2015 [27]	Canada	To determine physicians’ educational needs regarding medical cannabis.	426 physicians (44% GPs/family physicians and 51% other specialists, 4% other) answered a questionnaire (response rate: 1%, calculated from reported numbers).	Experiences: 79% had been approached by a patient to discuss the use of medical cannabis, and 39% had initiated a discussion with the patient about it. 59% had ever prescribed medical cannabis. There was a gap between current and desired knowledge concerning dosing, development and treatment plans, and the physicians reported a need for better knowledge of risks and benefits. Attitudes and beliefs: 65% were concerned that patients who requested medical cannabis actually wanted it for recreational purposes. The majority of physicians reported that no other health professionals than physicians should be authorised to approve medical cannabis.	See the chapter “Procedures and definitions of medical cannabis prescriptions in included studies”.
Fitzcharles 2014 [28]	Canada	To examine confidence in the knowledge of cannabinoids among rheumatologists.	128 rheumatologists answered a questionnaire (response rate: 25%). The study compared responders who are confident and not confident in knowledge of cannabinoids.	Experiences: 13% had ever recommended medical cannabis, and 90% were not confident in writing a prescription when required to indicate dosing, frequency, and method of administration. The study found that 7% of the not-confident vs. 27% of the confident rheumatologists had previously recommended a trial of medical cannabis (*p* = 0.006). Attitudes and beliefs: 25% believed there was a role for medical cannabis in the management of rheumatic diseases. Barriers which would prevent physicians from prescribing medical cannabis were history/potential of drug abuse/addiction, mental health and lack of clear diagnosis/non-severity of pain complaints. 42% of the confident physicians would prescribe medical cannabis if all conventional treatments had failed, which only 23% of the non-confident physicians would.	See the chapter “Procedures and definitions of medical cannabis prescriptions in included studies”.
Crowley 2017 [29]	Ireland	To assess levels of support for use of medical cannabis among GPs.	565 GPs answered a questionnaire (response rate 15%). The study compares GPs with and without special training in addiction treatment.	Attitudes and beliefs: Almost 60% supported legislation of cannabis for medical use. GPs with training in addiction treatment were significantly less supportive that cannabis should be legalised for medical use compares to GPs with no training (65.1% vs. 78.5% respectively (*p* = 0.003)). GPs with training in addiction treatment were significantly less supportive that medical cannabis has a role in pain management (80.1% vs. 88.2%, *p* = 0.03), treatment of multiple sclerosis (85.5 vs. 92.4%, *p* = 0.04), and palliative care (83.3% vs. 90.0%, *p* = 0.05), compared to GPs with no training.	See the chapter “Procedures and definitions of medical cannabis prescriptions in included studies”.
Van Hout 2017 [30]	Ireland	To study levels of support for medical cannabis among GPs.	565 GPs answered a qualitative survey with open-ended questions (response rate: 15%).	Attitudes and beliefs: The identified attitudes were mixed and centred on the evidence base and quality control. Some physicians were convinced about effects but concerned about the lack of evidence and patient misuse.	See the chapter “Procedures and definitions of medical cannabis prescriptions in included studies”.
Sharon 2018 [31]	Israel	To examine the attitudes, beliefs, and personal experience of pain specialists (physicians) in Israel regarding the medical use of cannabis.	50 registered, active, board-certified pain specialists answered a web-based questionnaire (response rate: 64%).	Experiences: 64% of all practicing pain specialists in Israel responded. 95% of them prescribed medical cannabis. Among them, 63% found cannabis moderately to highly effective, 56% have encountered mild or no side effects, and only 5% perceived it as significantly harmful. Common indications were neuropathic pain (65%), oncological pain (50%), arthralgias (25%), and any intractable pain (29%). Leading contraindications were schizophrenia (76%), pregnancy/breastfeeding (65%), and age < 18 years (59%). Attitudes and beliefs: Only 12% rated medical cannabis as more hazardous than opiates. 54% would like to see cannabis legalized in Israel. The majority (80%) reported they were inadequately trained to prescribe medical cannabis.	See the chapter “Procedures and definitions of medical cannabis prescriptions in included studies”.
Zolotov 2018 [9]	Israel	To study physicians’ views of medical cannabis and its possible integration into clinical practice.	Qualitative interviews with 24 physicians (specialists or physicians currently specializing in oncology, pain medicine, and family medicine) were conducted.	Attitudes and beliefs: The study identified two major narratives: 1) Cannabis as non-medicine: Conventional medicine was seen as the ideal option and physicians pointed out that medical cannabis failed to comply with the standards of biomedicine. Physicians pointed out the lack of scientific evidence of safety and efficacy. They presented medical cannabis as a social and criminal matter, which should not fall under the professional domain of medicine. 2) Cannabis as medicine: Hands-on experience was mentioned as having a crucial impact on attitudes and practices. Physicians pointed out the limitations of evidence-based medicine and argued that other aspects of health care are unsupported by evidence. When considering very sick people, physicians gave much less weight to the lack of evidence and potential harms. Besides, cannabis was described as medicine for patients with an uncontested diagnosis which can be proved by objective laboratory procedures.	See the chapter “Procedures and definitions of medical cannabis prescriptions in included studies”.
Ablin 2016 [32]	Israel	To survey rheumatologists’ attitudes towards the use of medical cannabis.	23 rheumatologists answered a questionnaire (response rate of 19.3%).	Experiences: 48% had previously prescribed medical cannabis, and 78% were not confident in writing prescriptions when required to indicate dosing, frequency and method of administration. Attitudes and beliefs: 83% were willing to prescribe medical cannabis if conventional treatments failed, and 9% would prescribe it regardless of previous treatments.	See the chapter “Procedures and definitions of medical cannabis prescriptions in included studies”.
Ebert 2015 [33]	Israel	To examine physicians’ experiences, knowledge and attitudes towards medical cannabis.	27 physicians (various specialities: oncology, pain medicine, rehabilitation, psychiatry and neurology) answered a questionnaire (response rate of 72%). The study compared physicians who had ever recommended medical cannabis with those who had not.	Experiences: About 90% have been exposed to a patient using medical cannabis, and 60% had recommended it to at least one patient. Compared to physicians who had never recommended medical cannabis (40.3%), physicians who had recommended it once or more (59.7) reported themselves as having a significantly greater knowledge of dosage, ways of administration and risk and adverse effects (*p* < 0.05). Clinical experiences and knowledge from medical literature and other physicians were influencing physicians’ decision on recommendation of medical cannabis. Attitudes and beliefs: About 80% reported that medical cannabis could be helpful for chronically and terminally ill patients. About 60% reported that physicians should not be permitted to prescribe it to patients directly, without the Ministry of Health’s licensing procedure. 88% reported a need for more education on medical cannabis.	See the chapter “Procedures and definitions of medical cannabis prescriptions in included studies”.

As our objective was to synthesize all existing literature about hospital physicians’ and GPs’ experiences, attitudes, and beliefs towards the use of medical cannabis, we did not perform a critical appraisal of the individual studies included. However, small study populations and low response rates are considered to be compromising for the validity of the study findings.

### Procedures and definitions of medical cannabis prescriptions in included studies

In the United States (US), Canada, Australia and Israel, medical cannabis was legal, but the physicians’ role in facilitating access varied. In none of the studies, physicians could provide medical cannabis to patients directly. In Ireland medical cannabis was illegal. In the US in general, medical cannabis was illegal according to the federal law, yet an increasing number of states have state-wise legalised medical use of cannabis [1]. All US studies included were conducted in such states. Physicians in states having authorised the medical use of cannabis could certify or recommend that their patients had a qualifying medical condition allowing the use of cannabis for medical purposes, but could not actually issue a prescription. The only lawful ways to dispense it, is as part of a federally approved research program or through state laws, which may permit caregivers and/or other healthcare workers to manufacture and distribute cannabis preparations to patients [34]. At the time when the studies were conducted, Canadian physicians could sign a document attesting that all conventional treatments had been tried and provide information on daily dose and duration of validity. Health Canada officials should subsequently give their approval [32]. In Israel, the use of medical cannabis was legal in terms of a licensing procedure, which meant that physicians could sign a medical recommendation, which was then processed and acknowledged by the Ministry of Health [1].

The terms used for the physicians’ procedure of facilitating access to medical cannabis are not consistent in the included articles; hence, regardless of the terms used in the individual studies, ‘the term ‘provide’ was chosen to be consistently used throughout this review, because it both relates to the issue of prescriptions as well as other ways of supplying patients with medical cannabis.

### Physicians’ experiences with patients receiving medical cannabis

#### Experiences with patient inquiries and prescriptions

Physicians experience inquiries about medical cannabis from a variety of patients, and some physicians provide it to their patients. The proportion of physicians having experienced inquiries about medical cannabis from patients varies between 49 and 95% in the identified studies [18–20, 24, 27]. The percentage of physicians reporting to have provided cannabis varies from 10 to 95% [15, 16, 19, 20, 22, 26–28, 31, 33, 35]. Especially three studies conducted in Israel report high proportions of physicians experienced in prescribing medical cannabis, namely 48, 60 and 95% respectively [31, 33, 35].

Seventy eight percent of physicians, feel uncomfortable with indicating dosage, frequency, and method of administration of cannabis prescriptions [35]. Studies show significant associations between physicians’ experiences with prescribing medical cannabis and their self-reported knowledge of it and confidence in prescribing it. Significantly higher proportions of physicians experienced in prescribing medical cannabis feel comfortable with providing it [26, 28] and report themselves as having greater knowledge of medical cannabis compared to physicians who have never provided it [33]. Additionally, another Israeli study finds that 60% of physicians report that they would not be willing to provide medical cannabis, without the licensing procedure at the Ministry of Health. This means that the ministry needs to approve a medical recommendation signed by the physicians, before the medical cannabis can be provided to the patient [33].

#### Experiences with effects, adverse effects, and misuse

In general, physicians experience a lack of knowledge about medical cannabis (64–90%) including beneficial as well as adverse effects [16, 20, 24, 25, 27, 28, 31, 35], and they do not feel confident using it in treatment of patients [24]. Despite this, many physicians (46–95%) still choose to provide it [16, 24, 31]. A qualitative interview study including physicians’ experiences with effects and adverse effects from medical cannabis, reported that some physicians, including family physicians and oncologists describe positive impressions of how medical cannabis helps their patients, and they view it as useful. They claim to see more efficacy of medical cannabis in real life than proven in literature, and hands-on experiences are mentioned as having a crucial impact on their views and on their decisions of providing it [9]. Similarly, in two recently published questionnaire-based studies, high proportions of physicians experience positive effects (63–67%) and mild or no side effects (56%) when using medical cannabis in treatment of certain ailments [16, 31]. The respondents in both of these studies are supportive of medical cannabis, and the authors concurrently conclude that the positive attitude may stem from the fact that the physicians’ patients experience beneficial effects of treatment with the medical cannabis.

### Physicians’ attitudes to and beliefs in medical cannabis

#### Attitudes towards prescription

There are various attitudes towards the prescription of medical cannabis among physicians. Some physicians argue that cannabis is a social and criminal matter which should not fall under the professional domain of medicine [9]. Other physicians accept cannabis as medicine for patients with a specific diagnosis [9].

.Legislation on the use of medical cannabis is widely discussed in the literature, and physicians are typically asked to report their opinion [21, 23, 29]. Studies generally show that significantly lower proportions of physicians with specialty, or other educational skills in addiction medicine, support the legal use of medical cannabis, compared to physicians with other specialties, including general practitioners (36% vs. 60%) [23, 29]. Mathern et al. investigated attitudes towards the use of medical cannabis in epilepsy patients and showed that a minority of epileptologists and general neurologists supported the use of medical cannabis for this group of patients [21].

#### Beliefs in effects, adverse effects, and misuse

Different beliefs in effects and adverse effects following clinical use of medical cannabis are reported among physicians. Conventional medicine is often seen as the ideal, and physicians argue that medical cannabis fails to comply with the standards of biomedicine [9, 30]. They point out the lack of scientific evidence of safety and efficacy [9, 30]. However, some physicians agree on the limited evidence, but argue that other aspects of health care are likewise unsupported by evidence [9]. When physicians consider treatment of pain and palliative care where all conventional methods have been tried, they give much less weight to the lack of evidence and potential harms and are more inclined to support the use of medical cannabis [9, 17, 28, 30, 33, 35]. Finally, some physicians are generally concerned that patients who request medical cannabis may actually want it for recreational use [27, 30].

An American study reports that almost 90% of its study population (palliative and hospice care providers) believe that cannabis can be useful in the treatment of pain, nausea, and appetite loss, and more than half of them believe that adverse effects are the same or less problematic than conventional treatments when considering pain, appetite loss, nausea, sleep, and end-of-life care [17].

Furthermore, hospital physicians’ and GPs’ beliefs in effectiveness seem to depend on their experiences and educational skills. Research shows that GPs experienced in providing medical cannabis are more convinced about benefits and less worried about harmful adverse effects compared to physicians who have never provided it (see Table 4) [22]. Accordingly, a recent American study find a high proportion of physicians willing to provide medical cannabis if it was legal (71% as an oral form, 86% as a topical treatment) [18]. An Irish study shows significant associations between GPs’ knowledge of substance misuse and their beliefs about efficiency, as a significantly smaller proportion of physicians with special training in addiction treatment believe that medical cannabis plays a role in pain treatment and palliative care, compared to physicians without this specialist training (see Table 4) [29].

## Discussion

### Statement of principal findings

This review shows that GPs and hospital physicians in various specialties often experience inquiries about medical cannabis from their patients and that they are willing to provide it to some degree. Although it should be noted that the number of prescribing physicians varies considerably, depending on setting, specialty, and experience among the physicians. However, physicians generally experience a lack of knowledge of clinical effects, both beneficial and adverse effects. Regarding physicians’ attitudes to and beliefs in medical cannabis, this review shows that physicians experienced in prescribing medical cannabis are more convinced of its benefits and less worried about adverse effects than physicians without experience. However, physicians specialized in addiction treatment and certain relevant indication areas seem to be more sceptical about using it for treatment of patients, compared to physicians in general.

### Strengths and weaknesses of the study

The strength of this review lies in the systematic approach to identifying peer-reviewed studies. However, most of the included studies were small and limited by relatively low response rates, which compromises the validity of the study findings (Tables 3 and 4). It is possible that responders differ from non-responders, as responders may be more interested in the topic and have more knowledge of it than non-responders. Besides, non-responders may have chosen not to participate, because they do not provide medical cannabis. This limits the generalisability of results of the single studies as well as this review.

Consequently, the heterogeneous study populations and patient groups limit the comparability of results. Experiences with and attitudes towards medical cannabis may vary as to specialty and the type of patients whom physicians see and treat in their daily work life, which makes study results less comparable. On the other hand, the heterogeneous sample also allows for the presentation of apparent differences between specialties and physicians. Yet, it could also be considered as a strength that the focus is on hospital physicians and GPs alone, unlike a recent review encompassing many types of healthcare professionals regardless of substantial differences in their right to make decisions about treatment of patients [10]. However, the laws on prescription of medical cannabis vary among the studied populations and between countries. This may affect the results between the individual studies and make them less comparable. Additionally, all the quantitative studies included used cross-sectional designs, which limits our possibility of concluding on causality, including facilitators and barriers, to prescribing medical cannabis.

The restrictions to the language of eligible publications may potentially have limited our study results, as articles published in other languages may present experiences and attitudes from physicians that cannot be generalized to the countries that were represented in this review. However, due to limited resources we were not able to translate and include these articles.

### Meaning of the study

This review shows that hospital physicians and GPs experienced in prescribing medical cannabis are more convinced about its benefits and less worried about adverse effects. This indicates that physicians have provided cannabis because they are confident about its effects. It is, however, possible that physicians to some degree report to be convinced of effects to justify their prescription. Furthermore, positive hands-on experiences with providing medical cannabis were described as having a crucial impact on their views, and the physicians’ experiences may be a facilitator for providing medical cannabis.

Generally, physicians with specialty in epileptology and neurology did not support treatment of epilepsy patients with medical cannabis [21], and those with specialty in addiction treatment or similar indication areas seemed to be more sceptical compared to other medical professionals [29]. This indicates that hospital physicians’ and GPs’ attitudes to and beliefs in medical cannabis are associated with their specialty, which might be explained by their different perspectives and experiences with patients’ needs, as well as their responsibilities for specific patient groups, e.g. patients who have substance abuse problems versus alleviation of pain in palliative patients. Physicians specialized in addiction treatment may mainly experience the adverse health effects from recreational use of cannabis, which possibly gives rise to their scepticism. However, these results are based on very few and small studies, and to gain deeper and more substantiated knowledge of possible associations between speciality training and prescription of medical cannabis more research is needed.

Regarding attitudes and beliefs, the results of this study are in line with another recent review [10]. However, this study adds new knowledge with its additional focus on actual experiences with providing it to patients. Hence, high proportions of hospital physicians and GP’s experienced in prescribing medical cannabis were found in the Israeli studies, which might be explained by the fact that Israel has had a less restrictive legal attitude towards cannabis use compared with the USA and Europe [32, 36] and has been running a medical cannabis programme since the late 1990s [37]. In addition, we found that hospital physicians’ and GPs’ experiences with prescriptions of medical cannabis were associated with their attitudes towards prescription and beliefs in effects. This may indicate that, over time, physicians and patients may become experienced in using medical cannabis, and that it gradually becomes more used and common. However, more studies focusing on such changes over time are needed to investigate, if the number of years during which medical cannabis has been an opportunity is influential on hospital physicians’ and GPs’ attitudes and beliefs.

### Unanswered questions and future research

This review shows a need for studies using stronger data collection methods to obtain larger study populations and reduce selection bias. Moreover, in the literature search, only two qualitative interview studies were identified, which also emphasizes the need for more qualitative studies on this topic to gain a deeper understanding of physicians’ attitudes, experiences, clinical practices, and the factors influencing this. It is reasonable to assume that the use of medical cannabis will increase and expand to more indication areas, as it gets legalised in more countries, and more physicians and patients become familiar with it. Continuous research in this area is needed to increase evidence about effect of medical cannabis and keep awareness of the facilitators and barriers to use medical cannabis as well as potentially induced harm.

## Conclusions

This review indicates that hospital physicians and GPs in various specialties experience inquiries about medical cannabis from their patients, and to some extent show openness to provide it, although there was a wide gap between studies in terms of willingness to provide. Most hospital physicians’ and GPs’ experience a lack of knowledge of beneficial effects, adverse effects and of how to advise patients. Hence, hospital physicians’ and GPs’ experiences with prescribing medical cannabis, and their knowledge as well as their specialty, may be associated with their attitudes towards prescription and beliefs in effect. More research, including larger studies with cohort designs and qualitative studies, is needed to examine facilitators and barriers to physicians’ prescribing practices.

## Data Availability

All study data are included in Table 4.
